# Antimicrobial activity of *Monascus purpureus*-derived red pigments against *Salmonella typhimurium*, *Escherichia coli*, and *Enterococcus faecalis*

**DOI:** 10.1186/s13568-024-01801-5

**Published:** 2025-01-04

**Authors:** Islam I. Teiba, Islam Mamdouh, Mokhtar I. Yousef, Ahmed Hussein, Emad H. El-Bilawy

**Affiliations:** 1 Microbiology, Botany Department, Faculty of Agriculture, Tanta University, Tanta City, 31527 Egypt; 2 Faculty of Basic Sciences, King Salman International University, South Sinai City, 46612 Egypt; 3 Department of Environmental Studies, Institute of Graduate Studies and Research, Alexandria University, Alexandria, 21526 Egypt; 4 Biotechnology Department Institute of Graduate Studies and Research, Alexandria University, Alexandria, 21526 Egypt

**Keywords:** Antimicrobial resistance (AMR), Bactericidal activity, Foodborne pathogens, Membrane permeability, Minimum inhibitory concentration (MIC)

## Abstract

The rise of antimicrobial-resistant microorganisms (AMR) poses a significant global challenge to human health and economic stability. In response, various scientific communities are seeking safe alternatives to antibiotics. This study comprehensively investigates the antibacterial effects of red dye derived from *Monascus purpureus* against three bacterial pathogens: *Salmonella typhimurium* ATCC14028, *Escherichia coli* ATCC8739, and *Enterococcus faecalis* ATCC25923. The dye was extracted from the *Monascus purpureus* ATCC16436 strain, using 1 mg of red dye in 1 ml of DMSO to achieve a concentration of 1000 µg/ml. The chemical profile of the red dye extract was analyzed using GC–MS analysis, confirming the presence of several bioactive antimicrobial compounds, including aspidospermidin-17-ol, 1-acetyl-16-methoxy, octanoic acid, and hexadecanoic acid methyl ester. The extract was tested against the bacterial strains at varying concentrations to determine the minimum inhibitory concentrations (MIC) and minimum bactericidal concentrations (MBC). The results demonstrated significant antibacterial activity, with the highest MIC and MBC values of 6.25/12.5 µg/ml against *S. typhimurium*. The antibacterial activity of the red dye was compared to five conventional antibiotics using the disc diffusion method, revealing superior effectiveness, particularly against *S. typhimurium*, with an inhibition zone measuring 20 ± 0.22 mm. Scanning electron microscopy was employed to explore the mechanism of action of the red dye extract, highlighting its impact on bacterial plasma membrane permeability and its interference with cellular energy production. These findings suggest that the *Monascus purpureus*-derived red dye extract represents a promising natural alternative to conventional antibiotics, demonstrating potent antibacterial activity and potential as a novel therapeutic agent in combating antimicrobial resistance.

## Introduction

Antimicrobial resistance (AMR) is a critical issue identified by the World Health Organization (WHO) as “a global public health concern” (Aslam et al. 2018). AMR has severe implications globally, contributing to increased morbidity and mortality from bacterial infections and necessitating urgent actions to address the issue (Abdelaziz Abdelmoneim et al. 2024). Over the past three decades, the approval rate of new antibiotics has declined, while antibiotic-resistant bacterial pathogens continue to emerge (Aslam et al. 2018). Furthermore, these pathogens are increasingly acquiring additional resistance mechanisms, resulting in the rise of multidrug-resistant (MDR), extensively drug-resistant (XDR), and pan drug-resistant (PDR) bacteria, which are resistant to all available antibiotics (Maarouf et al. 2023). While AMR mainly arises from the use of antibiotics and antimicrobials, substantial evidence indicates that its proliferation is also driven by poor local sanitation, pollution, and various non-usage factors, with the natural environment playing a significant role in its spread (Collignon et al. 2018; Graham et al. 2019). Living organisms participate in a complex network of interactions with their environment, maintaining a dynamic balance essential for life on Earth. Disruptions to this equilibrium can significantly harm ecosystems and the species within them (Teiba et al. 2024a, b). Microorganisms are found everywhere in the biosphere and play a crucial role in shaping their environments with their effects being beneficial, harmful, or sometimes subtle (Prosser et al. 2007). Despite the positive contributions of bacteria to human life and other organisms, certain bacterial species are responsible for numerous diseases in humans, animals, and other living beings. These pathogenic bacteria can cause a wide range of illnesses, from mild fevers to life-threatening conditions (MOFEED ET AL. 2022).

*Salmonella typhimurium*, a rod-shaped bacterium, poses a significant risk to human health as a leading cause of foodborne illness (Pławińska-Czarnak et al. 2023). Known as salmonellosis, this illness manifests with gastrointestinal symptoms such as diarrhea, abdominal cramps, and fever (Ali et al. 2024). Unlike its relative *Salmonella typhi*, which causes typhoid fever and invades the bloodstream, *S. typhimurium* typically results in less severe disease but can cause severe infections in young children, the elderly, and immunocompromised individuals (Crump and Mintz 2010; Scallan et al. 2011). Contaminated poultry products are a primary source of infection, highlighting the importance of proper food handling and cooking practices (Shaji et al. 2023).

*Escherichia coli* has a dual relationship with its host (Gambushe et al. 2022). While many strains coexist harmlessly in the intestines of humans and animals, aiding digestion (Flint et al. 2012), pathogenic strains can cause systemic infections due to specific virulence factors (Pokharel et al. 2023). These pathogenic strains disrupt the digestive system by producing toxins, leading to symptoms like abdominal cramps, diarrhea, and vomiting (Amemiya et al. 2023). Food contamination remains a common transmission route, underscoring the need for stringent hygiene practices to prevent infection (Denamur et al. 2021).

*Enterococcus faecalis* functions as both a commensal and a pathogen, with its pathogenicity often linked to host vulnerability, excessive intestinal growth, or medical devices (Archambaud et al. 2024; Repoila et al. 2022). Although traditionally considered an extracellular pathogen, *E. faecalis* can adhere to and enter mammalian cells, albeit less efficiently than intracellular enteropathogens, relying on carbohydrate-mediated adhesion (Ayobami et al. 2020; Comerlato et al. 2013). It is a leading cause of enterococcal infections, including healthcare-associated infections such as urinary tract infections, bacteremia, and endocarditis (Ben Braïek and Smaoui 2019). The emergence of antibiotic-resistant strains makes *E. faecalis* infections particularly challenging to treat (Boccella et al. 2021).

These three bacterial species highlight the concerning increase in antibiotic resistance and underscore the critical need for proper hygiene and food handling to prevent infections. The growing concerns about antibiotic overuse have spurred interest in sustainable, environmentally friendly alternatives derived from biological sources (El Basuini et al. 2024, 2022).

Natural food colorants are generally considered significantly better than synthetic dyes, which can sometimes be toxic and may trigger various allergic reactions and intolerances upon ingestion (Chaudhary et al. 2024). Additionally, many natural biopigments offer health benefits, including anti-cancer, antimicrobial, and therapeutic properties. They also often function as antioxidants, contributing further to human health (Chaudhary et al. 2022; Kiki 2023). In East Asian countries, *Monascus* pigments have been used as natural food colorants and additives for centuries. Produced by various *Monascus* species, these pigments can improve the color and sensory attributes of food (Haque et al. 2016). Additionally, due to their perceived health benefits, *Monascus* pigments are also being considered for medicinal use.

Microorganisms, especially the fungus *Monascus purpureus*, have been extensively utilized to produce natural red biopigments in response to growing consumer demand. *M. purpureus*, a filamentous fungus, is highly adaptable and generates various bioactive compounds, including flavonoids, phenols, tannins, and other secondary metabolites like biopigments (Kaur et al. 2023). Research has shown that consuming fermented rice with *Monascus purpureus* may help manage cholesterol levels, diabetes, cardiovascular diseases, and may even aid in cancer prevention (Kalaivani et al. 2010).

While *Monascus purpureus* red dye is well-known for its effectiveness as a food colorant and its potential benefits in human and animal medicine, its antimicrobial mechanisms are still not well understood. Therefore, this study aims to explore the antibacterial effects of red dye and its mode of action against three specific bacterial species: *S. typhimurium*, *E. coli*, and *E. faecalis*.

## Materials and methods

### Source and extraction of red dye

#### The source fungal strain

The *Monascus purpureus* strain ATCC16436 utilized in this study was obtained from the Microbiological Resources Center (MIRCEN) in Cairo, Egypt, which is part of the Egyptian microbial culture collection (ENCC), recognized for its ability to produce red pigments.

#### Composition of fungal culture media and culture properties

Potato dextrose agar was employed for the activation and short-term preservation of the fungal strain. To prepare the modified minimal medium (Embaby et al. 2018; Hamdiyati et al. 2016), 4 mL was combined with 3.0 g of rice grains per 100 mL. The medium consisted of 0.5 g each of NH_4_SO_4_, NH_4_NO_3_, KNO_3_, and peptone, along with 0.2 g of KH_2_PO_4_, K_2_HPO_4_, 0.2 g of MgSO_4_, 0.001 mM of ZnSO_4_, and 0.002 mM of MnSO_4_. This modified minimal medium served as the primary medium for the biosynthesis of *Monascus* pigments, with the final pH adjusted to (4.5). Potato dextrose agar plates were inoculated with fungal spores and then incubated in a static incubator (JSGI-100T, Korea) at 30 °C for 7 days.

### Extraction of red dye

A suspension of the fungal spores was prepared using sterile water, which was subsequently diluted and adjusted to a concentration of 2 × 10^4^ using a hemacytometer. *Monascus* pigments were extracted using a slightly modified procedure based on previous reports (Babitha et al. 2007). At the end of the incubation period, 20 mL of 96% ethanol was added to the fermented cake, and the mixture was incubated for 2 h while being agitated at 180 rpm. After incubation, the mixture was filtered through Whatman paper #1.0. The resulting filtrate was then centrifuged at 10,000 rpm for 10 min. The supernatant was collected and stored at 4 °C for further processing. For obtaining extract for antibacterial activity test, 1 mg of red dye was extracted using 1 ml of DMSO to obtain 1000 µg/ml solution.

### Chemical profiling of the red dye

Chemical profiling of the red dye sample was conducted using a Trace GC-TSQ mass spectrometer (Thermo Scientific, USA) equipped with a direct capillary column TG–5MS (30 m × 0.25 mm × 0.25 µm film thickness). The column oven temperature was initially set at 50 °C, then raised at a rate of 5 °C/min to 250 °C, where it was held for 2 min. It was further increased to a final temperature of 300 °C at a rate of 30 °C/min and held for another 2 min. The injector and MS transfer line temperatures were maintained at 270 °C and 260 °C, respectively. Helium was used as the carrier gas at a constant flow rate of 1 mL/min. The solvent delay was set to 4 min, and 1 µL of diluted samples was injected automatically using an autosampler AS1300 in split mode. EI mass spectra were recorded at an ionization voltage of 70 eV over the range of m/z 50–650 in full scan mode, with the ion source temperature set at 200 °C. Components were identified by comparing their mass spectra to those in the WILEY 09 and NIST 14 mass spectral databases.

### Anti-bacterial activity

#### Bacterial strains

The antibacterial activity of the red dye extract was assessed against three bacterial strains: *S. typhimurium* ATCC 14028, *E. coli* ATCC8739, and *Enterococcus faecalis* ATCC 25923, which were obtained from the Global Bioresource Center (ATCC, USA).

#### Minimum inhibitory concentration (MIC) and Bactericidal concentration (MBC)

The minimum inhibitory concentration (MIC) and minimum bactericidal concentration (MBC) were determined using 96-well microplates following the Clinical and Laboratory Standards Institute method (CLSI Ver. 2016 available at: https://asm.org/getattachment/2594ce26-bd44-47f6-8287-0657aa9185ad/Kirby-Bauer-Disk-Diffusion-Susceptibility-Test-Protocol-pdf.pdf) with a slight modifications according to Humphries et al. (2018). A bacterial inoculum of 1.5 × 10^8^ CFU/mL was added to each well. Various concentrations of the red dye extract were prepared in DMSO (200, 100, 50, 25, 12.5, 6.25, 3.125, 1.562, 0.781, and 0.390 μg/mL) and tested against the three bacterial strains in Mueller–Hinton broth (Oxoid, USA). For each assay, three wells containing bacterial suspension without the red dye served as growth controls, while three additional wells without bacterial inoculum acted as background controls. The optical density (O.D.) was measured at 620 nm using a microtiter plate reader (Thermo Scientific, USA). The MIC values were determined as the concentrations that inhibited over 95% of bacterial growth, while the MBC values were identified as those that killed more than 99% of the bacterial strains.

#### Comparison of antibacterial activity of red dye and conventional antibiotics

The comparison was conducted using the disc diffusion method following the protocol established by Surendra et al. (2011). Pure bacterial cultures were obtained by culturing the tested organisms in 1.5 mL of brain heart infusion broth (Oxoid, USA). After 24 h of incubation at 37 °C, the strains were streaked onto brain heart infusion agar for an additional 24 h. Several colonies from the solid culture were then inoculated into 10 mL of sterile saline solution to achieve a cell concentration of 0.5 McFarland standard (1.5 × 10^5^ CFU/mL). A sterile cotton swab was used to evenly spread the bacterial cultures on Mueller–Hinton agar (MH, Oxoid, USA) plates.

Cellulose discs (6.3 mm diameter) were impregnated with red dye at the highest concentration used in this study (200 μg/mL) and placed on the agar surface. The test was performed in triplicate. After a 24-h incubation period at 37 °C, the plates were examined, and the inhibition zones were measured to determine the average inhibition zone.

Additionally, antibiotic discs including ciprofloxacin (CIP; 5 µg), azithromycin (AZM; 15 µg), streptomycin (S; 10 µg), ampicillin-sulbactam (A/S; 10/10 µg), and clarithromycin (CLR; 15 µg) were added to the plates. The plates were incubated again at 37 °C for 24 h, after which the inhibition zones were measured, and the results were reported as the mean inhibition zone in millimeters.

#### Cellular structure of the tested bacterial strains

To investigate malformations in bacterial cellular structures, the tested bacteria were inoculated in nutrient broth (NB) media (Merck Millipore, Germany) containing red dye extract at a final concentration of 200 μg/mL and incubated at 37 °C for 12 h, with control samples included. Following incubation, the bacteria were centrifuged and fixed using a formalin-glutaraldehyde fixative (4F1G) in 0.1 M phosphate buffer (pH 7.4). The bacterial specimens underwent further fixation with 1% osmium tetroxide in 0.1 M phosphate buffer (pH 7.4).

Subsequently, the fixed specimens were dehydrated through a series of acetone concentrations. The specimens were then coated with gold–palladium using a Polaron E500 sputter coater (Polaron Equipment Ltd., England). Finally, the bacterial specimens were examined using a scanning electron microscope (JEOL JSM 35C).

### Statistical analysis

Statistical analysis was performed using one-way analysis of variance (one-way ANOVA) followed by Duncan's multiple range test. All experiments were conducted in triplicate, with statistical significance set at *p* < 0.01. Data was analyzed using SPSS® software (Version 27) to identify significant differences among means across all analyses.

## Results

### Chemical profiling of red dye

**Fig. 1:**
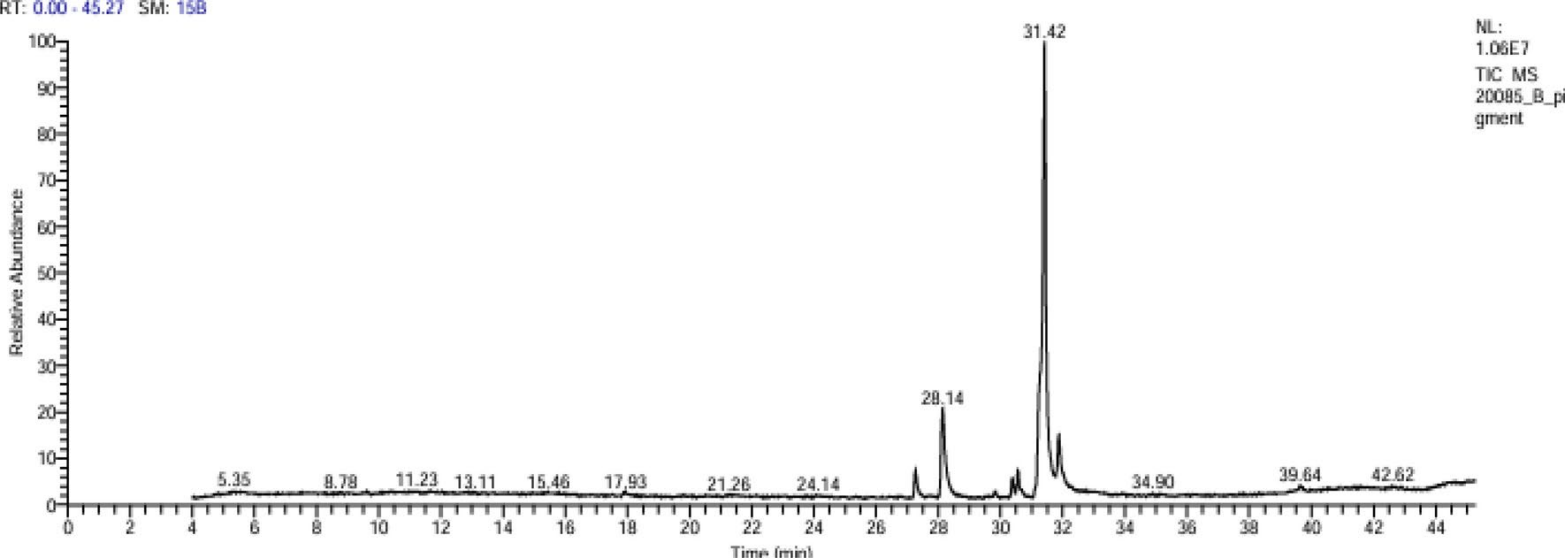
GC–MS chromatogram of *Monascus purpureus*-derived red dye extract

The chemical analysis of the red dye extract was performed using the GC–MS method. The obtained chromatogram is clearly illustrated in Fig. 1. A total of 24 molecules were identified in the red dye extract. Among the identified molecules, several bioactive compounds were well known for their different biological activities including antimicrobial, antiviral, antiprotozoal, and anticancer activities. The chemical molecules composition of the red dye with their IUPAC name, molecular formula, retention time, % area, and mass are shown in Table 1.

**Table 1:** Chemical profiling of *Monascus purpureus*-derived red dye extract

No	Compound	RT	%Area	Molecular formula	Mass
1	ASPIDOSPERMIDIN-17-OL,1-ACETYL-16-METHOXY-	5.23	0.32	C_22_H_30_N_2_O_3_	370
2	ETHANIMIDOTHIOIC ACID, 2-(DIMETHYLAMINO)-N-[[(METHYLAMINO)CARBONYL]OXY]-2-OXO-, METHYL ESTER	5.35	0.55	C_7_H_13_N_3_O_3_S	219
3	Octanoic acid, 7-oxo-	5.56	0.20	C_8_H_14_O_3_	158
4	Hexadecanoic acid, methyl ester	27.26	2.76	C_17_H_34_O_2_	270
5	n-Hexadecanoic acid	28.14	12.76	C_16_H_32_O_2_	256
6	17-Octadecynoic acid, TMS derivative	29.83	0.52	C_21_H_40_O_2_Si	352
7	9,12-Octadecadienoic acid, methylester, (E,E)-	30.38	1.55	C_19_H_34_O_2_	294
8	9-Octadecenoic acid (Z)-, methylester	30.56	2.10	C_19_H_36_O_2_	296
9	9-Octadecenoic acid (Z)-, methylester	30.67	0.64	C_19_H_36_O_2_	296
10	Oleic Acid	31.42	62.48	C_18_H_34_O_2_	288
11	Octadecanoic acid	31.87	10.38	C_18_H_36_O_2_	282
12	9-OCTADECENOIC ACID (Z)-	32.25	0.72	C_18_H_34_O_2_	282
13	9,12-Octadecadienoyl chloride, (Z,Z)-	32.32	0.69	C_18_H_31_ClO	298
14	HI-OLEIC SAFFLOWER OIL	32.46	0.44	C_21_H_22_O_11_	450
15	2-AMINOETHANETHIOL HYDROGEN SULFATE (ESTER)	32.53	0.38	C_2_H_7_NO_3_S_2_	157
16	13,16-Octadecadienoic acid, methylester	32.60	0.40	C19H34O2	294
17	17-Octadecynoic acid	32.72	0.54	C_18_H_32_O_2_	280
18	Cyclopentaneundecanoic acid	32.84	0.37	C_16_H_30_O_2_	254
19	8,11,14-Eicosatrienoic acid, (Z,Z,Z)-	33.03	0.37	C_20_H_34_O_2_	306
20	cis-5,8,11,14,17-Eicosapentaenoicacid	39.65	0.87	C_20_H_30_O_2_	302
21	9,12,15-OCTADECATRIENOIC ACID,	40.88	0.22	C_27_H_52_O_4_Si_2_	496
22	TRIDEUTERIOMETHYL10-EPOXY-7-ETHYL-3,11-DIMETHYLTRIDECA-2,6-DIENOATE	41.13	0.18	C_18_H_27_D_3_O_3_	297
23	1,25-Dihydroxyvitamin D3, TMSderivative	41.41	0.30	C_30_H_52_O_3_Si	488
24	CHOLEST-5-EN-3-YL(9Z)-9-OCTADECENOATE #	41.51	0.25	C_45_H_78_O_2_	650

### Antibacterial activity of red dye

The red dye extract demonstrated significant antibacterial effects against all three tested strains (Table 2). It showed the strongest activity against *S. typhimurium*, with minimum inhibitory concentration (MIC) and minimum bactericidal concentration (MBC) values of 6.25 and 12.5 µgml^−1^, respectively. The extract also exhibited considerable antibacterial activity against *E. faecalis*, with MIC and MBC values of 50 and 100 µgml^−1^. The weakest activity was observed against *E. coli*, both with MIC and MBC values of 100 µgml^−1^.

**Table 2:** The minimum inhibitory concentration (MIC) and minimum bactericidal concentration (MBC) of *Monascus purpureus*-derived red dye against the three tested bacterial strains

Bacterial Strain	MIC/MBC (µgml^−1)^
*S. typhimurium* ATCC14028	6.25/12.5
*E. coli* ATCC8739	100/100
*E. faecalis* ATCC25923	50/100

### Antibacterial activity of red dye against antibiotics

**Fig. 2:**
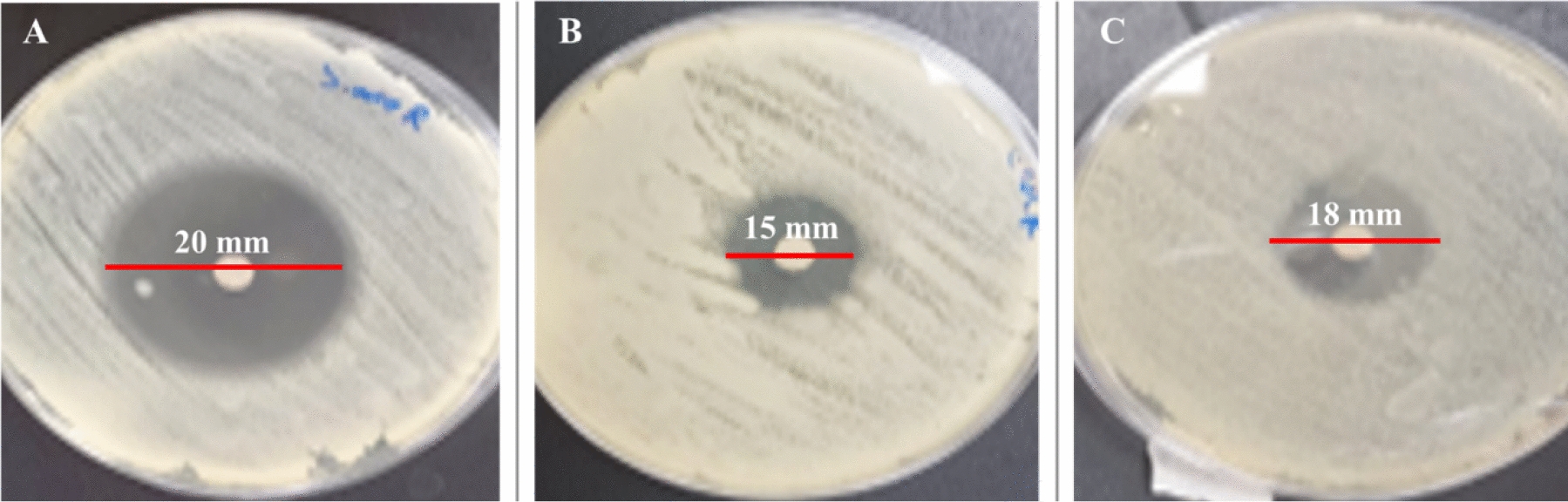
Antimicrobial activity of *Monascus purpureus*-derived red dye by disc diffusion method against: **A***Salmonella typhimurium* (zone of inhibition: 20 mm), **B***Escherichia coli* (zone of inhibition: 15 mm), and **C***Enterococcus faecalis* (zone of inhibition: 18 mm)

**Fig. 3:**
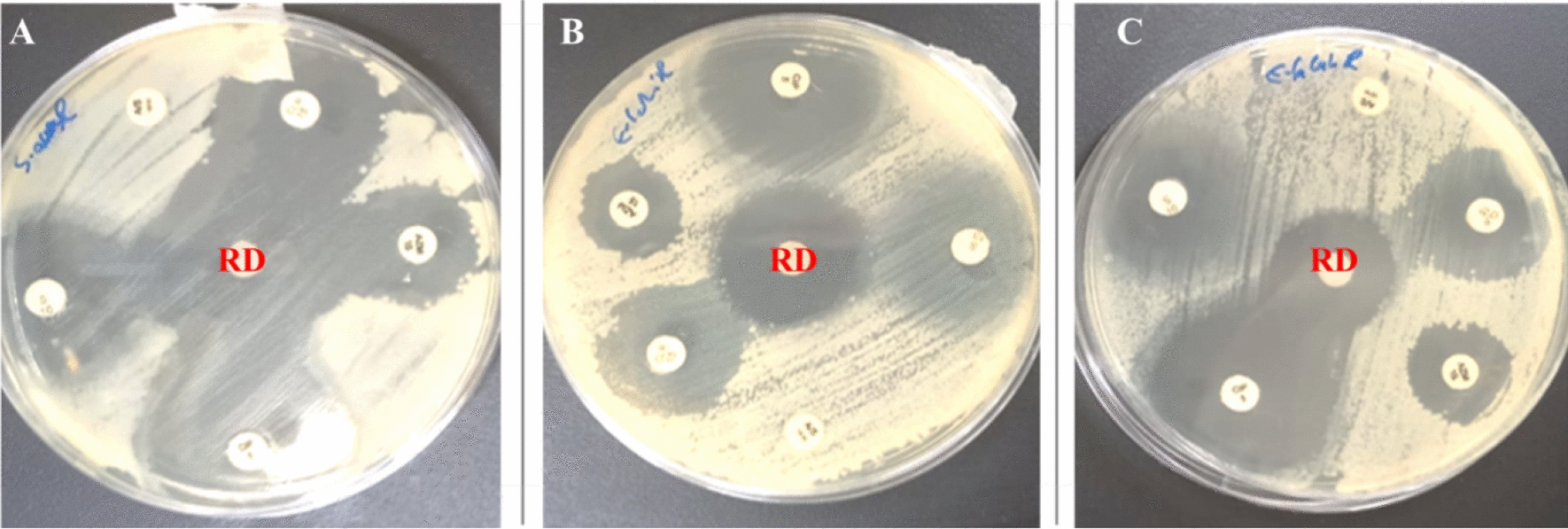
Zone of inhibition comparison between red dye (RD) and conventional antibiotics using disc diffusion method against: **A***Salmonella typhimurium*, **B***Escherichia coli*, and **C***Enterococcus faecalis*

Compared to traditionally used antibiotics, the red dye demonstrated a notable antibacterial effect against the tested strains (Fig. 2). The bacterial strains exhibited varying levels of sensitivity to the antibiotics used in this study (Fig. 3). Notably, the strains were only resistant to ampicillin-sulbactam. The findings are summarized in Table 3. The red dye extract demonstrated potent antibacterial activity against all tested strains, with inhibition zones ranging from 15 to 20 mm in diameter. The highest antimicrobial activity was observed against *S. typhimurium*, producing an inhibition zone of 20 ± 0.22 mm, followed by *E. faecalis* (18 ± 0.15 mm) and *E. coli* (15 ± 0.15 mm).

**Table 3:** The in vitro antibacterial activity of *Monascus purpureus*-derived red dye extract and conventional antibiotics was evaluated using the disc diffusion method. Results are presented as the mean inhibition zone in millimeters (mm) ± standard deviation (n = 3)

Bacterial strain	RD	CIP	AZM	S	A/S	CLR
*S. typhimurium* ATCC14028	20 ± 0.22	15 ± 0.08	8 ± 0.08	12 ± 0.15	R	11 ± 0.09
*E. coli* ATCC8739	15 ± 0.15	15 ± 0.2	7 ± 0.1	14 ± 0.11	R*	10 ± 0.08
*E. faecalis* ATCC25923	18 ± 0.15	18 ± 0.07	9 ± 0.06	17 ± 0.24	R	17 ± 0.21

A/S: ampicillin-sulbactam (10/10 µg); AZM: azithromycin (15 µg); CIP: ciprofloxacin (5 µg); CLR: clarithromycin (15 µg); R: resistant (No inhibition zone detected); RD: red dye; S: streptomycin (10 µg)

### Cellular structure of pathogenic strains

**Fig. 4:**
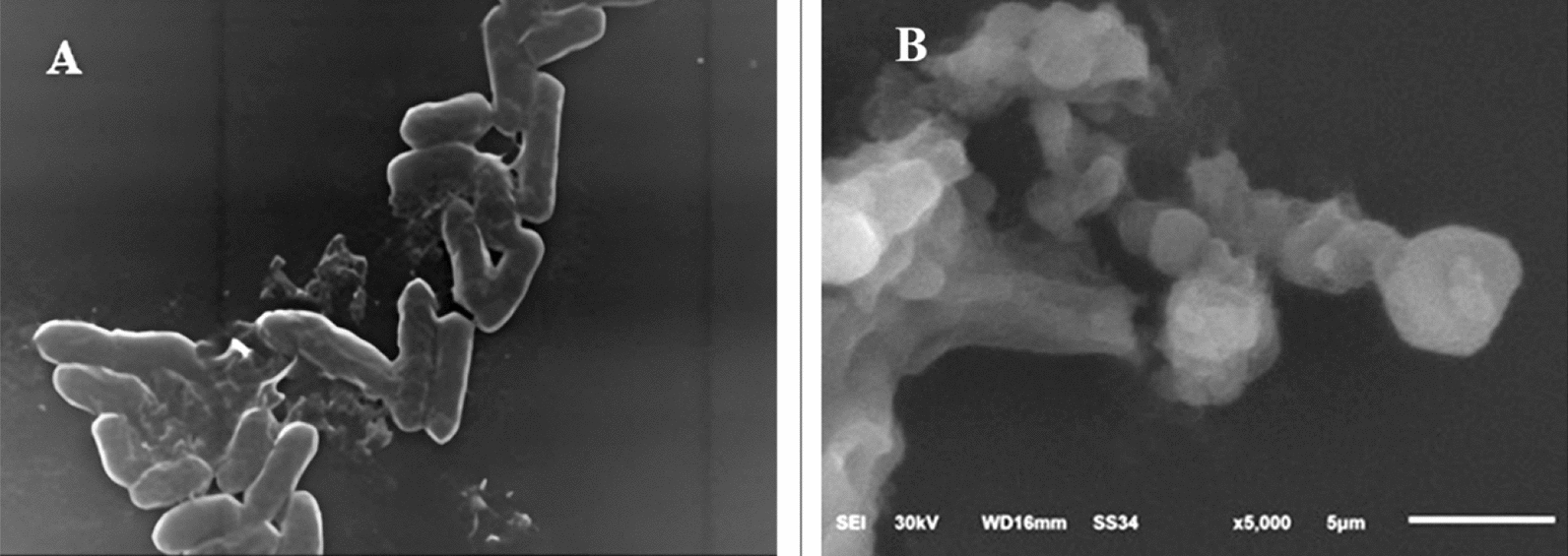
SEM micrographs illustrate the cytomorphology of *Salmonella typhimurium*: **A** before treatment (control) and **B** after treatment with *Monascus purpureus*-derived red dye extract

To thoroughly assess the bactericidal effect of the red dye on the three bacterial strains, their morphology was analyzed using scanning electron microscopy at the highest concentration of red dye (200 µgml^−1^) compared to the control. Control *S. typhimurium* cells were normally a rod-like bacillus shape with a smooth surface and a rigid, well-defined structure. After treatment with the red dye, these cells became enlarged and malformed, showing tiny holes and dents across most of the population (Fig. 4).

**Fig. 5:**
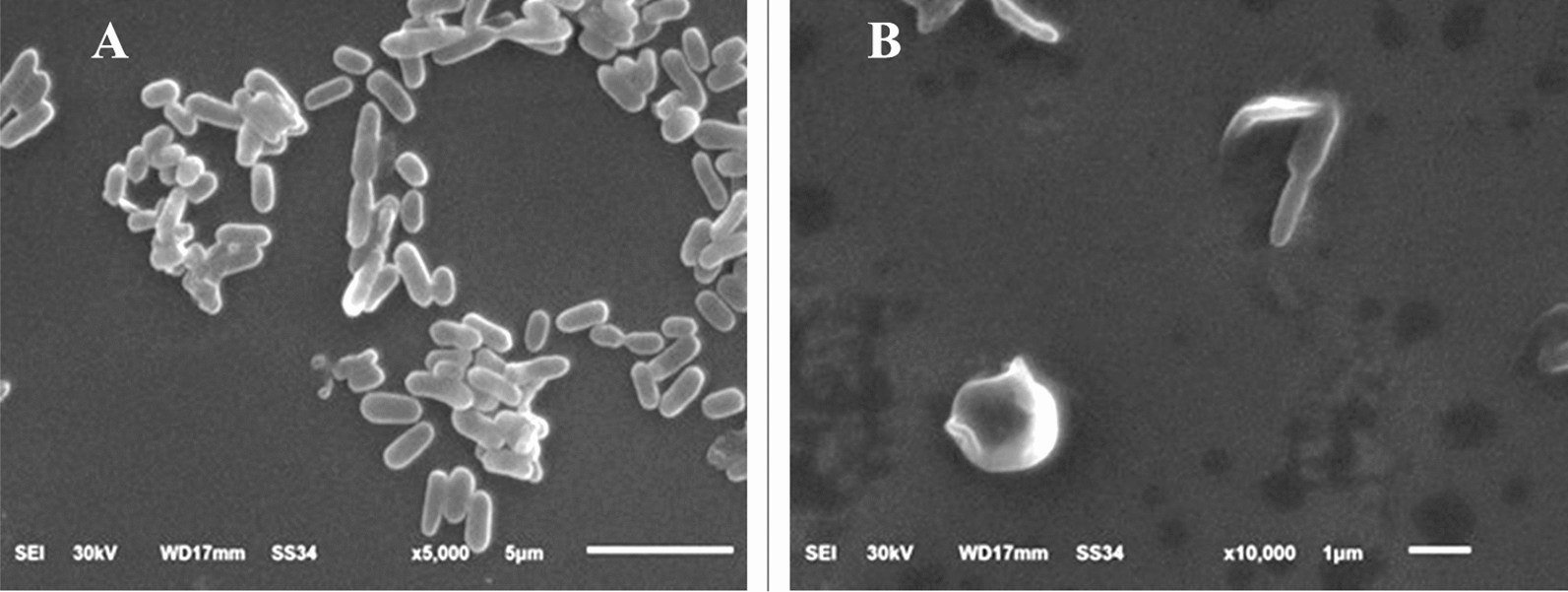
SEM micrographs illustrate the cytomorphology of *Escherichia coli*: **A** before treatment (control) and **B** after treatment with *Monascus purpureus*-derived red dye extract

Control *E. coli* cells appeared as normal rods with a smooth, well-defined surface. In contrast, the treated *E. coli* cells were predominantly degraded, exhibiting a ragged, shrunk, and wrinkled appearance, with noticeable dents and holes on their surfaces; some cells appeared hollow with empty ends (Fig. 5).

**Fig. 6:**
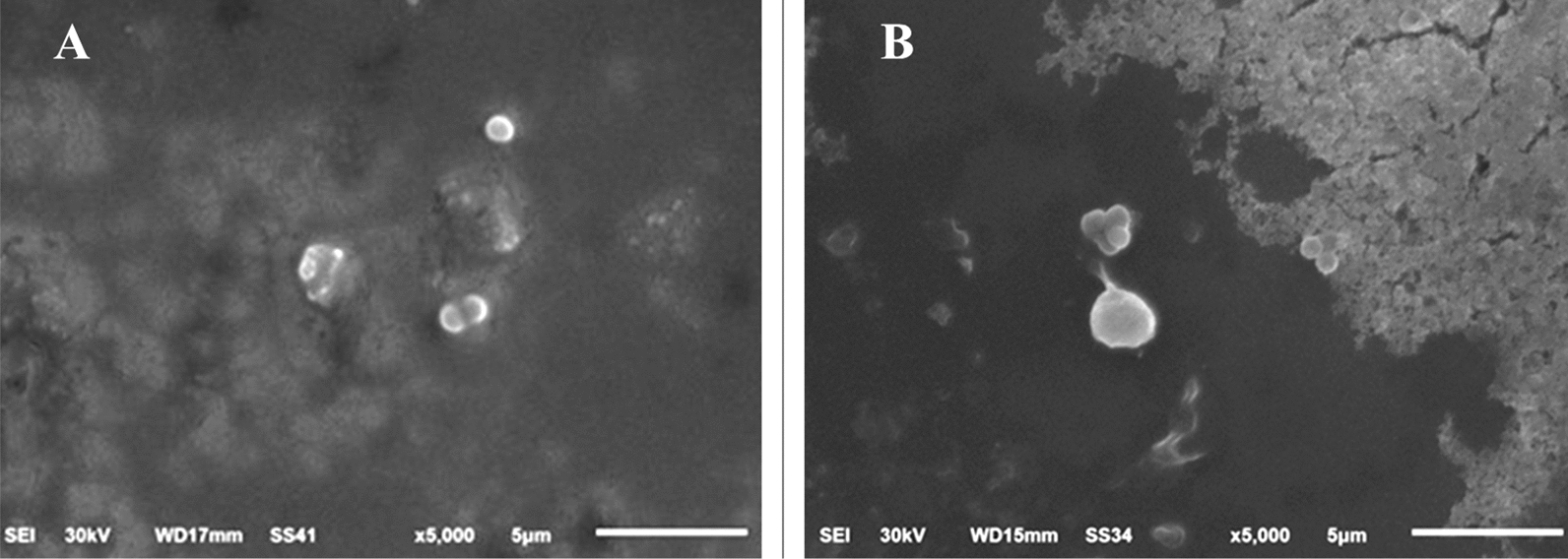
SEM micrographs illustrate the cytomorphology of *Enterococcus faecalis*: **A** before treatment (control) and **B** after treatment with *Monascus purpureus*-derived red dye extract

The control micrographs of *E. faecalis* revealed oval cells with smooth surfaces, typically arranged in pairs. However, treatment with the red dye caused malformation, resulting in shrunk and ragged cells with various tiny holes and dents on their surfaces (Fig. 6).

## Discussion

Antimicrobial resistance (AMR) refers to the ability of microorganisms to withstand the effects of various antimicrobial agents, particularly antibiotics. While AMR is a natural evolutionary process, the misuse and overuse of antibiotics has significantly exacerbated the problem (Tang et al. 2023). In 2019, AMR was directly linked to approximately 1.27 million deaths, with projections estimating over 10 million deaths annually by 2050 if current trends continue (Tang et al. 2023). In 2020, the WHO warned that without significant changes in antibiotic usage, AMR could evolve into a global health crisis comparable to a pandemic (Nieuwlaat et al. 2021).

Recently, researchers have increasingly focused on natural products as potential alternatives to traditional antibiotics (El Basuini et al. 2022). This study examines the red dye from *Monascus purpureus* as a potential antimicrobial agent, particularly against three bacterial pathogens. Biochemical analysis of the red dye identified a highly bioactive component. GC–MS profiling revealed various bioactive molecules, including aspidospermidin-17-ol, 1-acetyl-16-methoxy, octanoic acid, hexadecanoic acid methyl ester, and cyclopentaneundecanoic acid. These compounds are associated with broad-spectrum antimicrobial properties, as well as antiviral, anti-inflammatory, immunomodulatory, and anticancer activities; and some are used in commercially available therapeutics (Chaudhary et al. 2024; Wang et al. 2021).

The GC–MS analysis also indicated a high content of free fatty acids (FFAs), particularly oleic acid, which constituted approximately 62% of the chemical composition of the red dye extract. Free fatty acids are well-known for their antimicrobial, especially antibacterial, properties. Many organisms utilize the antibacterial effects of FFAs to protect themselves against parasitic or pathogenic bacteria (Desbois and Smith 2010).

In this study, the results from the minimum inhibitory concentration (MIC) and minimum bactericidal concentration (MBC) tests demonstrated strong antibacterial activity of the red dye against all three tested strains, with the dye particularly showing the most effectiveness against *S. typhimurium*. There is limited published data on the antibacterial activity of *Monascus* red dye against the specific bacterial species examined in this study. But similar antibacterial activity findings have been reported in previous studies, especially regarding *Bacillus subtilis* ATCC 6633 and *Escherichia coli* MG1655 (Husakova et al. 2024). Additionally, Chaudhary et al. (2022) demonstrated antibacterial activity of the *Monascus* red dye against *Bacillus cereus*, *Escherichia coli* at concentration of 200 µg ml.^−1^

The extracted red pigment exhibited substantial antimicrobial efficacy across all examined bacterial isolates, demonstrating inhibition zones with diameters ranging between 15 and 20 mm. Maximum growth inhibition was evidenced against *Salmonella typhimurium*, yielding a zone of inhibition measuring 20 ± 0.22 mm in diameter, followed by *Enterococcus faecalis* and *Escherichia coli*, displaying inhibition zones of 18 ± 0.15 mm and 15 ± 0.15 mm in diameter, respectively. When compared with five conventional antibiotics, the bacterial strains exhibited sensitivity to four antimicrobial agents but demonstrated complete resistance to ampicillin-sulbactam. Among the tested antibiotics, ciprofloxacin demonstrated the highest efficacy, while azithromycin showed minimal inhibitory effects. These findings align with previous investigations (Čujová et al. 2014; Tanuwidjaja et al. 2021), suggesting that the red dye extract could serve as a promising alternative antimicrobial agent, given its superior inhibitory effects compared to several conventional antibiotics.

To further investigate the impact of the red dye on bacterial cell structure, microscopic examinations were conducted. Electron micrographs of the tested strains compared to control cells revealed notable malformations in cytomorphology. The treated strains exhibited ragged, shrunken, and wrinkled appearances, in contrast to the well-defined, smooth surfaces of the control cells.

The SEM results provide valuable insights into the mechanism by which the red dye affects bacterial cells, indicating that it may increases membrane permeability and leads to ATP loss. This aligns with findings from the GC–MS profiling, which suggests that free fatty acids (FFAs) in the red dye disrupt cellular energy production (Alamsjah et al. 2009). The antibacterial action of FFAs may result also stem from several factors, including inhibition of enzyme activity, impairment of nutrient uptake, production of peroxidation and auto-oxidation degradation products, or direct lysis of bacterial cells (Desbois and Smith 2010; Drake et al. 2008).

In the context of escalating antimicrobial resistance (AMR), this study unveils the promising potential of *Monascus purpureus*-derived red dye as a versatile antimicrobial agent with broad applicational prospects. The extract's significant antibacterial efficacy against *Salmonella typhimurium*, *Enterococcus faecalis*, and *Escherichia coli*, coupled with superior inhibition compared to conventional antibiotics, suggests transformative implications across multiple domains. Beyond clinical applications, this natural compound holds substantial potential in agricultural settings for crop protection, livestock health management, and food preservation. The diverse bioactive metabolites identified through GC–MS analysis indicate broader applications in sustainable agriculture, potentially offering an eco-friendly alternative to synthetic antimicrobial interventions. Future research should explore its utility in developing natural biopesticides, enhancing livestock disease management strategies, and creating innovative food safety technologies, positioning this *Monascus purpureus*-derived red dye as a promising multifunctional solution in addressing contemporary challenges of microbial control across interdisciplinary fields.

## Data Availability

The authors confirm the data that support the findings of this paper are available from the authors upon request.

## Abbreviations

A/S: Ampicillin-sulbactam
AMR: Antimicrobial resistance
ATCC: American type culture collection
ATP: Adenosine triphosphate
AZM: Azithromycin
CIP: Ciprofloxacin
CLR: Clarithromycin
*E. coli*: *Escherichia coli*
*E. faecalis*: *Enterococcus faecalis*
ENCC: Egyptian microbial culture collection
FFAs: Free fatty acids
GC-MS: Gas chromatography-mass spectrometry
MBC: Minimum bactericidal concentration
MDR: Multidrug-resistant
MIC: Minimum inhibitory concentration
MIRCEN: Microbiological resources center
NB: Nutrient broth
PDR: Pan drug-resistant
S: Streptomycin
*S. typhimurium*: *Salmonella typhimurium*
SEM: Scanning electron microscopy
SPSS: Statistical package for the social sciences
WHO: World Health Organization
XDR: Extensively drug-resistant
